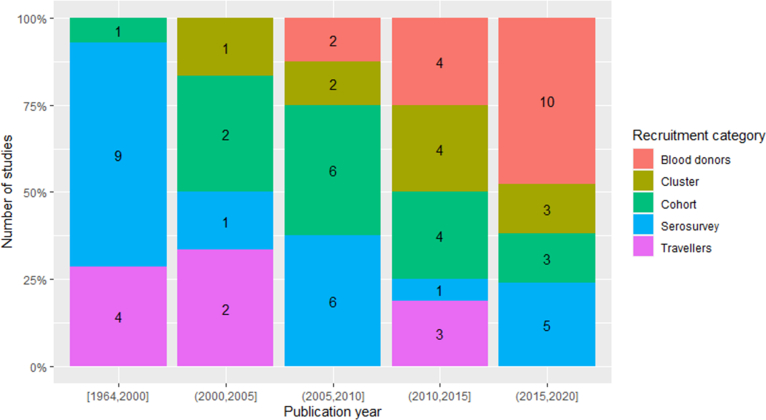# Asymptomatic dengue infection rate: A systematic literature review

**DOI:** 10.1016/j.heliyon.2023.e20069

**Published:** 2023-09-16

**Authors:** Olga De Santis, Nicolas Bouscaren, Antoine Flahault

**Affiliations:** a Institute of Global Health, Faculty of Medicine, University of Geneva, 1202 Geneva, Switzerland; b Direction de la recherche, de l'innovation et de la coopération internationale, CHU de La Réunion, 97410, Saint-Pierre, France; c Service de Santé Publique et Soutien à la Recherche, Inserm CIC1410, CHU de La Réunion, 97410 Saint-Pierre, France

**Keywords:** Dengue, Asymptomatic infection, Subclinical infection

## Abstract

**Objectives:**

Dengue infection is spreading worldwide. The clinical spectrum is broad and includes asymptomatic infections. This review provides an overview of the different proportions of asymptomatic infections described in epidemiological studies according to definitions, study designs, and detection methods.

**Methods:**

Medline and Embase databases were searched without restriction of date or language. Studies were included if they reported data on the incidence or prevalence of asymptomatic dengue infections. The data were summarized and classified according to the definitions of the term 'asymptomatic'.

**Results:**

A total of 74 studies were included. The mean proportion of asymptomatic infections among dengue-infected persons was 54% in 50 included studies. The prevalence of dengue infections detected in healthy persons was 0.2% in 24 included studies. The term ‘asymptomatic’ has been used to refer to ‘clinically undetectable infection’, but also to ‘undiagnosed infection’ or ‘mild infection’. Only 8% were clinically undetectable laboratory-confirmed dengue infections.

**Conclusion:**

The proportion of asymptomatic dengue infections varied greatly. Studies proving data on clinically undetectable laboratory-confirmed dengue infections were very few, but provided consistent results of low proportions of asymptomatic infections. These data challenge the assumption that the majority of dengue cases are asymptomatic.

## 1 Introduction

Dengue is the most globally prevalent arboviral disease [1]. Up to 3.97 billion people is at risk of contracting dengue virus infection [2]. Incidence of dengue has increased thirty times during the last fifty years. 50 to 100 million new infections are estimated to occur annually [1,3,4]. This increasing trend is expected to continue due to the increase in urbanization, population size, air traffic and climate change [5,6].

The symptomatic disease spectrum ranges from mild fever to severe and deadly haemorrhagic fever and shock syndrome [1]. The dengue-like syndrome is defined, according to the World Health Organization, as an acute fever disease with two or more of the following signs or symptoms: nausea, vomiting, rash, headache, retro-orbital pain, myalgia, arthralgia, and haemorrhagic signs [1].

A dengue virus infection can also present without clinically detectable symptoms. *Duong* et al. *in 2015* demonstrated that symptomless infected humans transmitted the virus to *Aedes aegypti* [7]*,* which could have a significant impact in spreading the disease. It is commonly accepted that the higher proportion of dengue infections are ‘*asymptomatic*’. The factsheet about dengue of the European Centre for Disease Prevention and Control mentioned: “*up to 40–80% of all dengue infections are asymptomatic*” [8]. However, data on the proportion of dengue infection referred to as ‘asymptomatic’ are highly heterogeneous in literature, which could be partly explained by a lack of clarity in the wording and on the meaning of the word ‘asymptomatic’. As illustrated by the WHO Key facts of the May 10, 2021 [8] “*A vast majority of cases are asymptomatic or mild and self-managed, and hence the actual numbers of dengue cases are under-reported*”, the clinical term ‘asymptomatic’ is frequently associated to ‘mild’ infections or ‘under-reported’ infections. Clinical terms such as ‘asymptomatic’ defined as the absence of symptoms, or ‘mild’ presentations are often gathered with the epidemiological consideration of ‘under-reported’ or ‘unapparent’ infections which correspond to undetected cases regardless of the presence or absence of symptoms. In fact, there are many reasons why dengue infection remains ‘unapparent’ for health authorities, apart from infections without symptoms, including mild symptoms, easily self-managed symptoms by an accustomed population, a lack of access to health care or misdiagnoses with other febrile diseases.

For epidemiological needs, such as mathematical modelling, infections presenting no symptoms are frequently pooled with ‘unapparent symptomatic’ infections as in *Clapham* et al. *2017* meta-analysis [9], where the word ‘asymptomatic’ gathers ‘clinically asymptomatic’ plus ‘unapparent symptomatic’ resulting in a proportion of 82% ‘asymptomatic’ for primary dengue infections and 59% for secondary infections in cohort studies and 78% and 43% respectively in cluster studies. These data are used for a mathematical modelling study [10] that concludes, “*more than 80% of dengue virus infections are attributable to individuals with mild to no symptoms who do not seek treatment from a physician*”. As described in these studies, for epidemiological and modelling purposes, dengue infections could be divided into infections with no symptoms, detected symptomatic infections and undetected symptomatic infections. Grange et al., in 2014 [11] published a comprehensive review of the epidemiological factors associated with the frequency of unapparent dengue virus infections. They concluded that the epidemiological evidence suggested that the majority of infections were unapparent in endemic regions and highlighted the important role of short-term immunity. In their review, they grouped together under the term “unapparent” infections that were clinically undetectable, mild and not detected by surveillance systems. In this review, we aimed to provide an update as many studies have been published since 2014, but also to offer a different perspective by trying to demonstrate that part of the large heterogeneity in rates of asymptomatic dengue between studies is due to a lack of standardisation of terminology and study designs, which jeopardises the assessment of associated risk factors.

The proportion of infections with no symptoms needs to be known and clearly distinguished from ‘unapparent’ grouped data, because a symptomatic infection, even a ‘mild’ one, can be detected by the patient him/herself or by a healthcare practitioner, using a questionnaire, whereas infections without any symptoms are completely hidden. So, when deciding on screening policies for blood donors or at the start of new outbreaks and on the preventive message to be communicated to the population, it is essential to distinguish between infections whose symptoms are not clinically detectable and ‘unapparent’ infections, both of which are described as ‘asymptomatic’.

The authors of this review recently conducted an observational study in the Southwest Indian Ocean islands [12], where dengue is emerging, to estimate the proportion of the infections with no symptoms through active screening for dengue infections in the community and, surprisingly, none could be found. Given the wide variation in data and the lack of clarity in clinical and epidemiological formulation in the literature, we decided to undertake this literature review to extract the proportions of ‘asymptomatic’ infections in epidemiological studies and to provide data with a higher granularity based on a specific definition of ‘asymptomatic’ and the detection method used.

## 2 Methods

### 2.1 Eligibility criteria

All observational studies reporting proportion, prevalence or incidence of asymptomatic dengue infections were included. No language, publication date, or publication status restrictions were imposed. Participants with any age with a diagnosis of dengue were considered.

A confirmed infection of dengue was defined by the detection of the virus with one of the following assays: virus culture and isolation, real-time polymerase chain reaction (RT-PCR), transcription-mediated amplification (TMA), detection of non-structural protein 1 antigen (NS1) by ELISA or rapid diagnostic tests (RDT); or, detection of an antibody response as a seroconversion or a 4-fold increase in total antibodies with one of the following assays: IgM/IgG ELISA or RDT, haemagglutination inhibition assays, plaque reduction neutralisation test (PRNT).

We did not restrict to a pre-defined definition of ‘asymptomatic infection’ nor to a pre-defined term used.

### 2.2 Information sources

Studies were identified by searching electronic databases and scanning references lists of published literature reviews on the topic. No limits were applied for language and other than English or French languages' paper were translated. This search was applied to the National Library of MEDicine's MEDLINE (1966-Present) by PubMed and Embase (1980-present) by ODS and NB. The last search was run on December 01, 2020. ODS and NB conducted the search in blind each other. In addition to searching databases, authors used i) https://connectedpapers.com/, ii) search in Google Scholar and iii) backward citation tracking to identify articles not retrieved by electronic searches.

### 2.3 Search

The following search strategies were used: Pubmed: ((dengue[MeSH Terms]) AND ((asymptomatic infection[MeSH Terms]) OR (asymptomatic disease[MeSH Terms]) OR (infection, subclinical[MeSH Terms]))); Embase: ‘dengue’ AND (‘asymptomatic infection’ OR ‘asymptomatic disease’).

Observational studies found through backward citation tracking were included if they contained data on the proportion of dengue asymptomatic infections.

### 2.4 Study selection

First, eligibility assessment was performed independently in an unblinded standardized manner by two reviewers (ODS and NB) by screening first title and abstract. If title and/or abstract provided insufficient information to assess the relevance or if a final decision could not be made, the full article was assessed. Second, full texts of articles selected in the first stage were independently reviewed for final inclusion. Disagreements were resolved by discussion between the two reviewers (ODS and NB). All duplicates’ articles were removed. When more than one published manuscript concerned the same study, these manuscripts were pooled, and data were extracted only once.

### 2.5 Data collection process

We developed a data extraction sheet containing the data items listed below. ODS extracted the data from included studies and NB checked the extracted data. Disagreements were resolved by discussion.

### 2.6 Data items

Data extracted concerned the following items: publication year, study site, recruitment design, age of participants, term used to describe ‘asymptomatic infections’, definition of ‘asymptomatic infection’, dengue diagnostic test, proportion of asymptomatic infections on total dengue infection, asymptomatic/symptomatic ratio, percent of dengue infection among asymptomatic participants, follow-up of dengue infection if any.

### 2.7 Risk of bias in individual studies

Risk of bias in individual studies were discussed considering the recruitment design and the diagnostic test used. We attempted to minimize selection, publication, and language bias through a comprehensive search strategy without language restrictions and by employing a transparent methodology. However, some biases remain due to the lack of standardization regarding the definition of “asymptomatic” infections and variations in recruitment designs among the included studies. The main bias encountered in this review, as explained later, is recall bias in serosurveys, particularly in children. In fact, as discussed later, the clinical presentation of dengue is similar to that of other common viral diseases, making it highly likely that children and parents may not remember the specific occurrence of a dengue infection.

### 2.8 Data analysis

R Core Team (2021) was used to calculate the summary statistics. The estimated proportions of asymptomatic infections and confidence intervals were obtained by compiling the frequencies of asymptomatic infections and the sample sizes of the populations of the different selected studies.

## 3 Results

### 3.1 Search strategy and PRISMA flow diagram

**Fig. 1:**
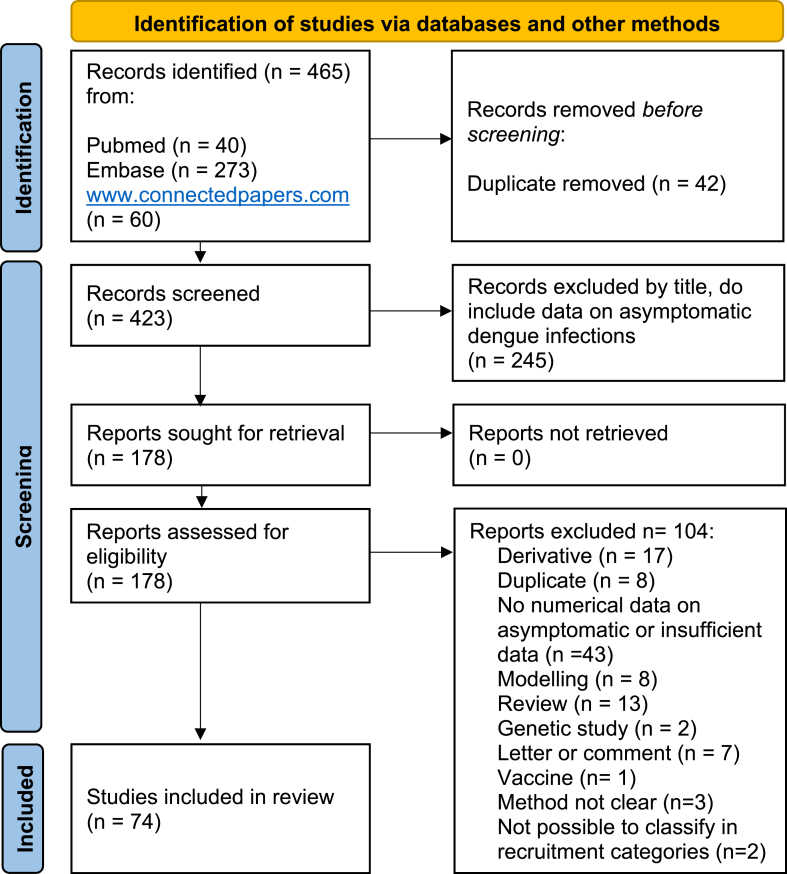
PRISMA Flow diagram.

We identified 465 papers. After an automatic removal of 42 duplicates by a reference management tool (Endnote), 423 papers were screened by title, of which 178 were selected, retrieved and assessed for eligibility, with abstract and full text reading. One hundred and four papers were excluded (reasons listed in Fig. 1: PRISMA flow diagram), and 74 were included, listed in Table S1 (supplementary material). Table 1 lists the 50 studies that provided asymptomatic rates of dengue infections by calculating the number of asymptomatic infection among the total dengue infections detected and the associated study characteristics and epidemiological risk factors. The 24 remaining studies, presented in Supplementary Table S1, provided the prevalence of dengue infections among asymptomatic populations.

*From:* Page MJ, McKenzie JE, Bossuyt PM, Boutron I, Hoffmann TC, Mulrow CD et al. The PRISMA 2020 statement: an updated guideline for reporting systematic reviews. BMJ 2021; 372:n71. https://doi.org/10.1136/bmj.n71. For more information, visit: http://www.prisma-statement.org/

**Table 1:** List of 50 observational studies providing data on the asymptomatic rate of dengue infections.

Definition	Reference	Publication Year	Study Type	Region	**Age (years)**	Diagnostic Test	Serotype	Sero-prevalence	Incidence*	**Dengue infections** N	**Asympto-matic Rate** % (N)
**No symptom**	[29]	2020	Cluster	South-East Asia	11–63	RT-PCR	Dengue virus 1 (1/13) Dengue virus 2 (2/13) Dengue virus 3 (7/13) Dengue virus 4 (5/13)	9%	NA	175	7.4% (13)
**No symptom**	[38]	2000	Travellers	America, Caribbean	≥ 14	IgM/IgG ELISA, PRNT	NA	69%	NA	22	0% (0)
**No symptoms**	[31,39,40]	2008	Cluster	South-East Asia	0.5–15	RT-PCR, IgM/IgG ELISA	All, mainly Dengue virus 1, Dengue virus 4	8%	NA	119	20% (24)
**No symptoms**	[41]	2010	Cluster	Latin America	All	IgM/IgG ELISA, HI titersx4, RT-PCR	Dengue virus 2	4%	NA	12	42% (5)
**No symptoms**	[14]	2018	Cluster	Latin America	All	RT-PCR, NS1	NA	13%	NA	50	32% (16)
**No symptoms**	[30,42]	2019	Cluster	South-East Asia	0.5–40	RT-PCR	Dengue virus 1 (80.8%) Dengue virus 2 (7.7%) Dengue virus 4 (11.5%) Dengue virus 3 (1.1%)	4%	NA	346	7.5% (26)
**Subclinical**	[43]	2015	Cluster	South-East Asia	All (mainly <15)	IgM/IgG ELISA	NA	6%	NA	113	92% (104)
**Subclinical**	[44]	2005	Cluster	South-East Asia	All	HI titersx4, RT-PCR	Dengue virus 1, Dengue virus 2	2%	NA	17	47% (8)
**Subclinical**	[45]	2012	Cluster	South-East Asa, Latin America	>2	RT-PCR	Dengue virus 1, Dengue virus 2, Dengue virus 3	10%	NA	101	29% (29)
**Subclinical**	[46]	2016	Cluster	South Asia	All	RDT NS1,IgM/IgG	NA	11%	NA	226	63% (142)
**Subclinical**	[17]	2015	Cluster	Latin America	≥ 5	IgM/IgG ELISA seronconversion or ab titers x4	NA	22%	NA	253	60% (151)
**Subclinical**	[47]	2015	Cluster	East Asia	All	IgM/igG ELISA	NA	5%	NA	41	68% (28)
**Subclinical**	[48]	2011	Cohort	South-East Asia	0–8	PRNTx4	NA	NA	11%	NA	75% (NA)
**Subclinical**	[15]	2016	Cohort	South-East Asia	All	HI titersx4	NA	NA	9%	77	79% (61)
**Subclinical**	[35]	2009	Cohort	South-East Asia	<1	IgM/IgG ELISA	NA	NA	1%	10	90% (9)
**Subclinical**	[49]	2010	Cohort	South-East Asia	2–15	IgG ELISA	Dengue virus 2, Dengue virus 1	NA	3%	953	80% (764)
**Subclinical**	[50]	2013	Cohort	Latin America	≥ 5	IgM/IgG ELISA, PRNT	Dengue virus 3, Dengue virus 4	NA	11%	2286	90% (2074)
**Subclinical**	[51]	1973	Serosurvey	Latin America	All	HI titersx4	NA	45%	NA	148	43% (63)
**Subclinical**	[52]	1967	Serosurvey	Latin America	>4	HI titers x4	NA	38%	NA	25	16% (4)
**Subclinical**	[53]	1985	Serosurvey	Latin America	All	HI titersx4	Dengue virus 4	7%	NA	56	45% (25)
**Subclinical**	[54]	2009	Serosurvey	Latin America	1–79	IgM/IgG ELISA	NA	10%	NA	33	70% (23)
**Subclinical**	[55]	1998	Serosurvey	Latin America	NA	HI	Dengue virus 2	44%	NA	588	41% (243)
**Subclinical**	[56]	1998	Serosurvey	Oceania	14–50	HI, IgG ELISA, PRNT	Dengue virus 2	26%	NA	139	11.5% (16)
**Subclinical**	[57]	1990	Serosurvey	Latin America	All	PRNT	Dengue virus 1, Dengue virus 2	17%	NA	219	76% (167)
**Subclinical**	[58]	2013	Serosurvey	South-East Asia	7–85	IgM/IgG ELISA	NA	7%	NA	NA	78% (NA)
**Subclinical**	[59]	2006	Serosurvey	East Asia	≥ 18	IgG ELISA	Dengue virus 2	NA	NA	55	78% (43)
**Subclinical**	[60]	2006	Serosurvey	Latin America	7–20	Viral isolation	Dengue virus 1, Dengue virus 2, Dengue virus 3, Dengue virus 4	7%	NA	215	86% (185)
**Subclinical**	[61]	2002	Serosurvey	Latin America	≥ 14	IgM/IgG ELISA	Dengue virus 1	21%	NA	42	33% (14)
**Subclinical**	[62]	2011	Travellers	America, Caribbean	≥ 18	RT-PCR, IgM ELISA	Dengue virus 1	33%	NA	7	0% (0)
**Subclinical**	[63]	2011	Travellers	NA	≥ 18	IgM/IgG ELISA	NA	1%	NA	14	64% (9)
**Subclinical**	[64]	2002	Travellers	NA	≥ 18	IgM/IgG ELISA	NA	3%	NA	NA	77% (NA)
**Subclinical**	[65]	1999	Travellers	NA	≥ 18	IgM/IgG ELISA	NA	7%	NA	7	42% (3)
**Subclinical**	[21]	2012	Travellers	Asia	≥ 16	IgG ELISA	NA	1%	NA	4	100% (4)
**Subclinical**	[66]	2005	Travellers	South-East Asia	≥ 18	Serology	NA	10%	NA	27	11% (3)
**Subclinical**	[26]	1969	Travellers	South-East Asia	All	Serology	NA	NA	NA	NA	0% (NA)
**Subclinical**	[67]	1995	Travellers	Horn of Africa	≥ 18	IgM ELISA	NA	9%	NA	44	16% (7)
**Unapparent**	[68]	1988	Cohort	South-East Asia	4–16	HI titers x4	Dengue virus 1, Dengue virus 2, Dengue virus 4	NA	12%	103	87% (90)
**Unapparent**	[69,70]	2005	Cohort	South-East Asia	18–66	HI titersx4	All, mainly Dengue virus 4	NA	1%	NA	72% (NA)
**Unapparent**	[16,34,71,72]	2002	Cohort	South-East Asia	7–16	HI titers x4	All, mainly Dengue virus 3	NA	7%	615	66% (406)
**Unapparent**	[73]	2006	Cohort	Latin America	4–16	HI titersx5	Dengue virus 1, Dengue virus 2	NA	5%	NA	85–92% (NA)
**Unapparent**	[74,75]	2010	Cohort	Latin America	2–14	Total antibodies Inhibition ELISA titersx4	Dengue virus 1, Dengue virus 2	NA	1%	NA	60–95% (NA)
**Unapparent**	[76]	2010	Cohort	South-East Asia	6 and 18 weeks	HI titersx4	NA	NA	1%	NA	85% (NA)
**Unapparent**	[77]	2015	Cohort	Latin America	10–18	IgG ELISA	NA	NA	6%	19	61% (10)
**Unapparent**	[78]	2010	Cohort	Latin America	5–60	PRNT	Dengue virus 1, Dengue virus 2, Dengue virus 3	NA	7%	NA	50–84% (NA)
**Unapparent**	[79]	2014	Cohort	South-East Asia	≤ 12	IgG ELISA	NA	NA	4%	67	60% (40)
**Unapparent**	[80]	1995	Serosurvey	Latin America	5–19	HI	Dengue virus 1, Dengue virus 2	62%	NA	277	58% (160)
**Unapparent**	[81]	1995	Serosurvey	Latin America	All	IgM/IgG ELISA	NA	17%	NA	59	53% (28)
**Unapparent**	[82]	2000	Serosurvey	Latin America	All	IgM ELISA, PRNT	Dengue virus 1, Dengue virus 2	41%	NA	NA	97% (NA)
**Unapparent**	[83]	2006	Serosurvey	South-East Asia	All (mainly >18)	IgM ELISA	NA	NA	21%	NA	82% (NA)
**Unapparent**	[84]	2009	Serosurvey	South-East Asia	18–74	IgM/IgG ELISA	NA	3%	NA	NA	96% (NA)

### 3.2 Study sites

Excluding three studies in travellers in multiples regions, 96% of studies took place in Asia and Latin America, half in each continent. Only one study was conducted in Africa. In supplementary material. As presented in Table 1, the Asymptomatic Rate (AR) per region ranged from 16 to 97% (19 studies, median 58%) for Latin America; 0–96% (19 studies, median 75%) for South-east Asia; 63–100% (4 studies) for East Asia; 0% (2 studies) for America, Caribbean; 16% (1 study) for the Horn of Africa and 11.5% (1 study) for Oceania.

### 3.3 Publication dates

Most of the studies (73%) were published since 2006. Before 2000, only some serosurveys were conducted in endemic areas or after outbreaks, and some studies on travellers returning from endemic countries. In the last fifteen years, cohort or cluster studies were mostly conducted. In the last five years, many studies on blood donors were published. (See Fig. S1 in Supplementary material).

Different meanings of ‘asymptomatic’ dengue infection.

We defined the three following categories to classify with more precision the group of dengue infections referred to as ‘asymptomatic’ detected by the included studies.

- the *“no symptoms”*: detected laboratory confirmed infections with absolutely no symptoms declared during a follow-up; 6 studies corresponds to this definition and 3/6 were published after the Grange et al., 2014 [11], previous review. The AR ranged from 0 to 42% (median 7.5%)
- the *“subclinical*”: mild or aspecific infections: presence of symptoms but that do not fit with the WHO definition of clinical dengue; 30 studies included with this definition. The AR ranged form 0–100% (median 64%)
- the *“unapparent”*: infections not detected by the health care system or by any surveillance system regardless of symptomatology; 14 studies. The AR ranged from 50 to 97% (median 72%)

The term ‘asymptomatic’ is kept as a container including all the above definitions and corresponds to the proportions of cases extracted in the studies because referred to as ‘asymptomatic’.

### 3.4 Proportion of asymptomatic participants among dengue infections (asymptomatic rate) versus prevalence of dengue infections among asymptomatic participants

In the majority of the included studies (50), the results extracted for this review were the proportions of asymptomatic participants among dengue infections. Studies were classified according to the definitions of ‘asymptomatic’, the diagnostic tests and the age group as shown in the summary Table 2. In the remaining 24 studies, including all the studies on blood donors (16), 2 cohorts and 6 serosurveys, the results presented were the prevalence of dengue infections among healthy participants. We presented these results separately in Table 2, classified according to the viral or antibody detection method. Indeed, in these 24 studies, the denominator is a population of healthy persons and not dengue infected cases. The 6 serosurveys studies presented in Table 2 presented a proportion of participants with traces of old dengue infections (i.e. presence of IgG in blood) but no dengue history. We decided to include these studies in the review, as they are another way to detect the presence of possible dengue infections with no symptoms, in the population. The studies on blood donors are important to evaluate the risk of dengue transmission through blood transfusion.

**Table 2:** Prevalence of dengue infection among asymptomatic participants per study category and detection method.

Recruitment method	Virus detection^b^					Antibody detection				
	Number of studies	References	I/A^a^	Prevalence of dengue infection (CI)	Range	Number of studies	References	I/A^a^	Prevalence of dengue infections (CI)	Range
**Blood donors**	14	[13,[85], [86], [87], [88], [89], [90], [91], [92], [93], [94], [95], [96], [97]]	125/54333	0,2% (0-0,7%)	[0–5.5%]	2	[98,99]	15/573	2,60% (0–6,8%)	[0–4.2%]
**Cohort**	1	[100]	NA	12,70%		1	[32]	NA	5–20%	
**Serosurvey**^c^						6	[33,[101], [102], [103], [104], [105]]	NA	28%	[7–48%]
**Total**	15					9				

a I/A: number of dengue infections (I) among healthy asymptomatic people (A).

b Virus detection thourgh RT-PCR, Transcription Mediated Amplification (TMA) or NS1.

c Presence of IgG and no history of dengue.

### 3.5 Asymptomatic rate according to the categories of recruitment and detection methods

Five categories according to the participants’ recruitment design were identified among the included studies: 1) Cluster studies; 2) Cohort studies; 3) Serosurveys; 4) Studies on travellers; 5) Studies on blood donors. Median (and interquartile range) proportions of asymptomatic infections according to the categories of recruitment for 50 studies providing data on the proportion of asymptomatic infection among dengue infections were calculated. Studies on travellers show the lower proportions of asymptomatic infections but with a high variability and cohort studies the higher with the shorter.

Each recruitment design presented different characteristics, risk of bias and distribution of dengue infections prevalence or incidence. Table 3 present the results of proportion of asymptomatic infections according to the clinical definition of ‘asymptomatic’ and to the detection method (viral or antibody). In cluster studies (number of studies = 11), the recruitment took place in geographical areas of a predefined radius, among people living in the neighbourhood of a dengue index case. Dengue prevalence in this kind of studies ranged from 2.2% to 21.5% (median 7.9%) and the AR ranged 7.4–92% (median 42%). Five studies provided proportions of asymptomatic infections corresponding to a strict clinical definition of ‘No symptom’ and detected with virus detection methods (RT-PCR or viral isolation), the mean proportion was of 8%.

**Table 3:** Summary table providing the proportions expressed in % of asymptomatic infections among dengue infections classified by the definition of ‘asymptomatic’ and the diagnostic test (the detailed tables are provided in supplementary material).

Recruitment category	Asymptomatic definition	Virus detection				Antibody detection			
		Number of studies	References	A/I^d^	Proportion of ‘asymptomatic’ infections (CI)	Number of studies	References	A/I^d^	Proportion of ‘asymptomatic’ infections (CI)
**Cluster**	No symptoms	5^a^	[14,29,31,41,42]	59/702	8% (5–12%)	2^a^	[31,41]	25/131	19% (10–28%)
	Subclinical	2^a^	[44,45]	31/118	26% (17–35%)	5^a^	[17,43,44,46,47]	433/650	67% (63–71%)
	Unapparent								
**Cohort**	No symptoms								
	Subclinical					5	[15,35,[48], [49], [50]]	2908/3326	87% (86–89%)
	Unapparent					9	[68,69,71,73,74,[76], [77], [78], [79]]	546/804^b^	68% (64–71%)
**Serosurvey**	No symptoms								
	Subclinical	1	[60]	185/215	86% (79–93%)	10	[[51], [52], [53], [54], [55], [56], [57], [58], [59],^61^]	598/1305	46% (43–49%)
	Unapparent					5	[[80], [81], [82], [83], [84]]	188/336^c^	56% (50–61%)
**Travellers**	No symptoms					1	[38]	0/22	0% (0–21%)
	Subclinical	1	[62]	0/7	0% (0–38%)	7	[21,26,[63], [64], [65], [66], [67]]	26/96	27% (17–37%)
	Unapparent								

a Ref 39, 47 and 53 used both detection methods: virus or antibody detection, results have been splitted for the table.

b Data extracted from 4/9 studies that provided detailed data.

c Data extracted from 2/5 studies that provied detailed data.

d Number of 'asymptomatic' dengue infection/total number of dengue infections, aggregated results extracted from the publications.

In cohort studies (n = 14), the recruitment took place in a predefined group of persons, as children of a primary school. Participants gave a blood sample at inclusion, and were followed by annual blood samples for serology. Detection of clinical apparent dengue infection was possible thanks to school absenteeism surveillance and with the collaboration of surrounding health centres. Dengue incidence ranged from 0.8% to 12.7% (median 6%) with an AR that ranged from 60 to 92% (median 78%). All cohort studies used antibody detection methods. By refining the results according to asymptomatic definition, only subclinical or unapparent infections were described with high proportion of 87 and 68% respectively (Table 2). The risk of recall bias was high mainly in children who might present aspecific clinical forms and would not have consulted a general practitioner for mild symptoms.

Serosurveys (n = 22) recruited participants in general population of an administrative region to know the attack rate and herd immunity of the population. Dengue prevalence showed a large distribution ranging from 2.6% to 61.6% (median 19.3%) with an AR range of 6.6–97% (median 53%). Sixteen serosurveys provided data on asymptomatic infections among dengue infections presented in Table 2. The proportion of asymptomatic infections according to clinical definition varies among 46–86%, only subclinical and unapparent infections were considered. Six serosurveys provided a mean of 28% of healthy participants who had IgG positive but no history of dengue infections (Table 1).The studies were retrospective with a high risk of recall bias. Interpretation of lab results differed among studies and the WHO recommendation to collect paired sera was not always respected.

Studies on travellers or migrants (n = 9) included adult participants, not immune for dengue virus, and not used to this disease. Sample sizes were small. Dengue prevalence showed also a large distribution among studies, between 1% and 68.8% (median 7.8%), the AR was of 0% for one study using virus detection method and between 0 and 27% for studies using antibody detection methods. The risk of recall bias was low as a symptomatic episode during a short holiday or mission was generally a key fact for participants. Moreover, the small size of the sample sizes and the very aggregated and defined group of participants allowed a better follow-up and accurate clinical data. Studies on travellers included almost only adult participants (one study >14 years old and another >16 years old), only one included also children. On seven studies including adult participants with available data, we gathered 125 adult participants. Twenty-six participants had subclinical symptoms. Thus 99/125 (79%) of adult travellers had typically dengue-like syndrome. Travellers all came from countries without dengue. In all but one study, the diagnostic was based on a seroconversion, which mean that all were primary infections.

Studies on blood donors (n = 16) only included adult ‘asymptomatic’ participants due to the eligibility criteria for blood donation. The prevalence of asymptomatic infections was among all the included study population and was equivalent to the prevalence of dengue infections that ranged between 0 and 5.5% (median 0.07%). As presented in Table 1, most of the studies (14/16) used viral detection methods and the prevalence of dengue were very low for all except for one study (5.5%) [13]. These studies were undertaken to assess the risk of dengue transmission through blood donation during outbreaks or in endemic countries but the methodology was not elaborated to detected and evaluate asymptomatic infections. Indeed, no questionnaire on signs and symptoms was submitted to the participants besides the basic eligibility criteria for blood donation. As no follow-up was undertaken, it was not possible to determine if a dengue-positive participant had an asymptomatic or a pre-symptomatic infection.

### 3.6 Asymptomatic rate according to the age group

By classifying the studies according to the age group (the age is mentioned in Table 1), it resulted that the AR for adults defined as, more than 14 years or more than 18 years old, depending on studies, ranged 0–96% (13 studies, median 33%). The AR for the children group ranged 20–87% (11 studies, median 66%) and two studies concerned infants (less than one year old) with an AR of 85–90%. The AR in 23 studies including participants of all ages ranged 0–97% (median 63%). The AR seems then be higher for children than for adults but with a high variability.

### 3.7 Asymptomatic rate according to the serotype

In Table 1 are listed the serotypes detected during the studies. Data were not available for 26 on 50 studies and when available, the data were mostly aggregated and did not provide sufficient detail to be able to calculate any association between the AR and the serotype circulated. Moreover, for many studies many or even all four serotypes were circulating concomitantly. We extracted the AR for the studies were one serotype was dominant: Dengue virus 1: AR 0–33% (4 studies); Dengue virus 2: AR 11.5–78% (4 studies); Dengue virus 3: AR 7.4–66% (2 studies); Dengue virus 4: AR 20–72% (3 studies). But here again, the variability is high and the number of studies low.

### 3.8 Asymptomatic rate according to primary versus secondary infections

Since the Grange et al. review [11], some new studies provided information concerning the association between the severity of symptoms and the immunity. Most of the recent studies suggest that primary infections are more likely to be overt symptomatic and milder or asymptomatic in secondary or repeated infections especially if the time between the infections is short [[14], [15], [16], [17]]. Inversely, Sun et Luo, China, 2018 [18], suggested that secondary or repeated infections are less likely to be asymptomatic.

## 4 Discussion

### 4.1 Main results

The mean proportion of ‘asymptomatic’ in the broad sense, including mild and unapparent dengue infections among identified dengue infections, was 54% overall in the 50 studies included in this review. By extracting data from studies with a precise definition of the word ‘asymptomatic’, meaning a clinical absence of symptoms, this proportion is equal to 18%. A proportion of 8% is obtained by combining the definition of no symptoms with a viral detection method using molecular detection, antigen detection or viral isolation. What is sometimes referred to as the ‘*majority*’ [19] or ‘*40*–*80%*’ [8,20] of asymptomatic dengue infections includes both purely no symptoms infections and subclinical or unapparent infections which could be confirmed infections detected by molecular biology but also suspected infection diagnosed by antibody detection.

The prevalence of dengue infections detected in apparent healthy participants in endemic countries was 14% in the 24 studies included. Including only blood donors and using viral detection methods yielded 0.2% of detected infections. If we exclude one study which presented particularly high results of 5.5% [13], the prevalence of dengue infections among blood donors, using the viral detection method, is 0.1%.

### 4.2 High heterogeneity in data

Our results showed that there was considerable heterogeneity in the proportion of dengue infections classified as ‘asymptomatic’ in the studies, which ranged from 0% to 100%. Methodological differences could explain this heterogeneity. The following parameters differed from one study to another: recruitment designs, the definitions considered for ‘asymptomatic’ infections, the age of the participants included and the diagnostic tests used to detect dengue infection. The extreme differences between 0 and 100% observed in the studies of travellers are also due to the small sample sizes and different interpretations of what was considered an ‘asymptomatic’ infection. For example, the study with 100% asymptomatic infections [21] included four dengue-infected travellers whose symptoms did not meet the WHO clinical definition of a dengue-like syndrome and who were therefore classified as ‘asymptomatic. In attempting to identify epidemiological risk factors – such as age, serotype, immunity, study location - associated to the AR of dengue infections, we were confronted with considerable heterogeneity in the results preventing to identify trends. This heterogeneity can be explained by study parameters that are not standardized, such as the definition considered for ‘asymptomatic’, the study recruitment design or the detection method. To be able to evaluate the risk factors more accurately, these parameter would have to be fixed, but there have not been enough studies published to have a reliable sample with fixed parameters. Up to now, only five studies shared the same definition of “no symptoms”, used a cluster recruitment design and an RT-PCR detection method.

### 4.3 Study sites

The study sites of almost all the included studies are located in Asia and in Latin America. Historically, the burden of dengue concerned essentially these two continents, sharing the same vector, *A. aegypti*. However, the epidemiology of dengue has changed, partly because of the spread of a second vector *A. albopictus* due to international trade [22,23]. *A. albopictus* invaded Africa since 1989 and was responsible for several dengue outbreaks [24]. Unfortunately, research on dengue has been completely neglected in this continent [25]. Although several studies were published on the epidemiology of dengue, the resulting knowledge is partial due to the exclusivity of study sites, which share a high level of endemicity and the same vector.

### 4.4 History of recruitment designs and asymptomatic detection throughout the years

This review includes 74 studies published between 1964 and 2020. Most of the studies (80%) were published in the last 20 years with an increasing trend over the last 5 years. Five categories of recruitment methods were identified: *Serosurveys in general population*, *Cohort studies*, *Cluster studies*, *Surveys on travellers or migrants* and *Surveys on blood donors*. Before 2001, all studies were serological surveys carried out in endemic countries or following outbreaks. Some were carried out on travellers returning from tropical area in European or US countries. The design of the studies subsequently changed to adapt and keep pace with the growing threat of dengue. The need for precise data on disease transmission and clinical presentations, led researchers to refine their methodologies by adopting cohort and cluster studies. Finally, recent years have seen an increase in studies of blood donors, reflecting the fear of this emerging disease and the need for data for policy makers.

The hypothesis that infections with no symptoms could play a role in the transmission of the disease appeared in the literature around 2000. Prior to this, a few sporadic detections of “*not overt diseases*” [26,27] had been described in large serosurveys. The increasing spread of the disease and the development of new vaccines [28] compelling to a better knowledge of the prevalence and herd immunity may explain the growing interest in studying infections without clinical presentation or not detected by surveillance systems.

### 4.5 Definitions of ‘asymptomatic’ in literature

No consensus has been reached on a standard definition or terminology for ‘asymptomatic’ dengue infections. The literature provided different terms such as: “*asymptomatic*”; “*subclinical*”; “*unapparent*” or “*inapparent*”; “*mild*”; “*not overt disease*”. These terms were not always defined in the same way and sometimes were used as synonyms. By refining the definitions and classifying the studies as *no symptoms*, *subclinical* and *unapparent*, the proportions of asymptomatic fall into opposite trends (18, 55 and 75% respectively).

The studies estimating the proportion of dengue infections with no symptoms detected by the presence of the virus (RT-PCR, TMA, virus isolation or NS1) were only five in number and resulted in a proportion of 8% of infections with no symptoms by aggregating the data. All five used a cluster recruitment design, which appears to be the most suitable for finding infections without any clinical presentation. The two studies with the largest sample sizes [7,29] were from Asia (Cambodia and Thailand) and both reached a same result of 7.5%.

### 4.6 Clinical presentation according to age

The high and prolonged endemicity meant that it was mainly children who fell victims to dengue and that by adulthood, they had already acquired partial or complete immunity. Most dengue infections were mild and rarely led to a medical consultation. The WHO has provided a clinical definition for a dengue-like syndrome but no definition for a “mild” dengue infection. In this review, the publications considered mild infections as infections without fever [30,31], or presenting low-grade dengue symptoms (headache, muscular and articular pain, rash, pruritus, fatigue) [32,33] or, mainly in children, as undifferentiated fevers presenting as other febrile childhood diseases [[34], [35], [36], [37]]. No typical symptoms that would help diagnose mild dengue infection emerged from the papers included in this review.

If we consider the studies carried out on travellers, 80% of these adult travellers with a primary dengue infection presented with a typical dengue-like syndrome. Twenty percent were not compatible with the WHO definition of dengue, which does not mean that they presented no symptoms. Only one study of travellers also included children and concluded that these children did not have typical dengue symptoms. In addition to travellers, most studies included participants with multiple dengue infections.

### 4.7 Parameters that contribute to explain the extent of asymptomatic infection in studies

By classifying the studies and extracting the data according to the following parameters: *recruitment design* - *definition of ‘asymptomatic’* - *age of participants* - *diagnostic tests* - it emerged that certain parameters appeared to influence the proportion of asymptomatic infections (see Table 2). This interpretation must be taken with caution, as the results could not be perfectly compared due to the lack of standardization in the methods used to collect the data. Nevertheless, it is possible to identify a trend in these factors that is medically and epidemiologically plausible. The following parameters appear to increase the proportion of asymptomatic infections: *“children”*, *“serosurveys”* and *“cohort studies”*, *“dengue diagnosed with antibodies detection”*. Rather, *“adults”*, *“cluster studies”, “studies on travellers”* and *“dengue diagnosed with viral detection methods”* would decrease this proportion.

The multiplication of study parameters such as methods of detection, study designs, and the clinical definition of “asymptomatic” makes it difficult or impossible to interpret the impact of other variables such as serotype or age on the rate of asymptomatic infections. However, the number of studies using a strict definition of the absence of symptoms, a similar design and a similar method of detection can be counted on the fingers of one hand (5). Further studies with fixed parameters are needed to determine the association of serotype, primary or secondary infection, and age on the proportion of asymptomatic cases.

### 4.8 Limitations and futures prospects

While the systematic review provides an overview of the proportions of asymptomatic dengue infections, it is subject to limitations mainly as heterogeneity of definitions, and variability in study designs. The lack of standardized criteria for defining asymptomatic dengue infections makes it challenging to compare and synthesize the results accurately. To address these limitations, future research should focus on establishing standardized definitions, conducting large-scale prospective studies, and strengthening global surveillance efforts. These steps will contribute to a more robust understanding of asymptomatic dengue infections and inform public health strategies to control and prevent the spread of the disease.

## 5 Conclusion

This literature review provides a more detailed understanding of the proportion of asymptomatic dengue infections. By carefully examining the available data and considering the context and design of the studies, it becomes evident that infections with no symptoms were rare. Most of the so-called ‘asymptomatic’ dengue infections were actually mild or nonspecific infections that could easily go undetected by public health surveillance. These considerations are particularly valuable for areas with high endemicity, as there is very little data available for geographic areas where dengue is emerging.

Dengue is rapidly spreading worldwide, reaching countries without herd immunity and where the population has limited knowledge about the disease. The transition to endemicity can occur swiftly, as demonstrated by the sustained transmission of the virus on La Reunion since 2016. Therefore, there is a critical need for data to model the disease and forecast its evolution; interpret surveillance data with caution, and discuss the possibility of introducing a vaccination. In areas recently affected by dengue, the proportion of infections with no symptoms appears to be quite low, as well as the proportion of unapparent cases, assuming adequate surveillance systems and the access to healthcare. However, if the disease become endemic, the proportion of asymptomatic infections, the persons most affected by the disease and the disease presentation may change.

Furthermore, this literature review highlights the lack of research and knowledge on the epidemiology of dengue in Africa and on the clinical presentation of dengue in countries where the disease is emerging. Most of the published data on clinical presentation focused on children, while information regarding the clinical presentation in the adult population was scarce. These data are crucial for assisting public health authorities in adapting policies related to dengue surveillance and blood donors, as well as determining the need introducing dengue vaccines.

## Ethical approval statement

Ethics approval was not required for this literature review.

## Author contribution statement

All authors listed have significantly contributed to the development and the writing of this article.

## Data availability statement

Data included in article/supplementary material/referenced in article.

## Declaration of competing interest

The authors declare the following financial interests/personal relationships which may be considered as potential competing interests:

Olga DE SANTIS reports financial support was provided by 10.13039/501100001711Swiss National Science Foundation. Olga DE SANTIS reports financial support was provided by the GlobalP3HS program for Global PhD Fellowship in Public Health Sciences funded by 10.13039/100018694Marie Sklodowska-Curie Actions (Horizon 2020-COFUND).

## Figures and Tables

**figs1:**